# A Rare Occurrence of Cutaneous Recurrence in a Patient of Post Modified Radical Mastectomy

**DOI:** 10.7759/cureus.16153

**Published:** 2021-07-04

**Authors:** Sri Hari Priya Vemulakonda, Naveen Kumar Gaur, Oseen Shaikh, Chellappa Vijayakumar, Uday Kumbhar

**Affiliations:** 1 Surgery, Jawaharlal Institute of Postgraduate Medical Education and Research, Puducherry, IND

**Keywords:** ductal carcinoma, chemotherapy, mastectomy, cutaneous recurrence, carcinoma breast

## Abstract

Carcinoma breast is the second most common malignancy in females. Due to the recent awareness and medical advances, most of the cases are diagnosed and treated at an early stage. Cutaneous recurrence without other distant metastasis post-surgery in carcinoma breast is usually uncommon. We report a 52-year-old lady who presented to us with cutaneous recurrence of carcinoma right breast, post neoadjuvant chemotherapy and modified radical mastectomy. The diagnosis was confirmed on histopathology after the biopsy from the cutaneous nodule. The patient was discussed in the tumor board and planned for palliative chemotherapy considering extensive cutaneous metastasis.

## Introduction

Skin metastasis is one of the rare clinical occurrences in visceral tumors. A very few percentages of the visceral tumors tend to metastasize to the skin [1]. The most common cancers that metastasize to the skin are melanoma, followed by breast. In females, around 20 percent more incidence of cutaneous metastasis is due to carcinoma breast than with other malignancies [2]. Cutaneous involvement either due to metastasis or recurrence indicates a very advanced stage of the disease with a grave prognosis. Diagnosis is usually clinical, and biopsy is confirmatory. Treatment of such presentation is complex and includes chemotherapy, palliative resection, and radiotherapy. We report a 52-year-old lady who had a cutaneous recurrence of carcinoma breast post neoadjuvant chemotherapy and modified radical mastectomy.

## Case presentation

**Figure 1:**
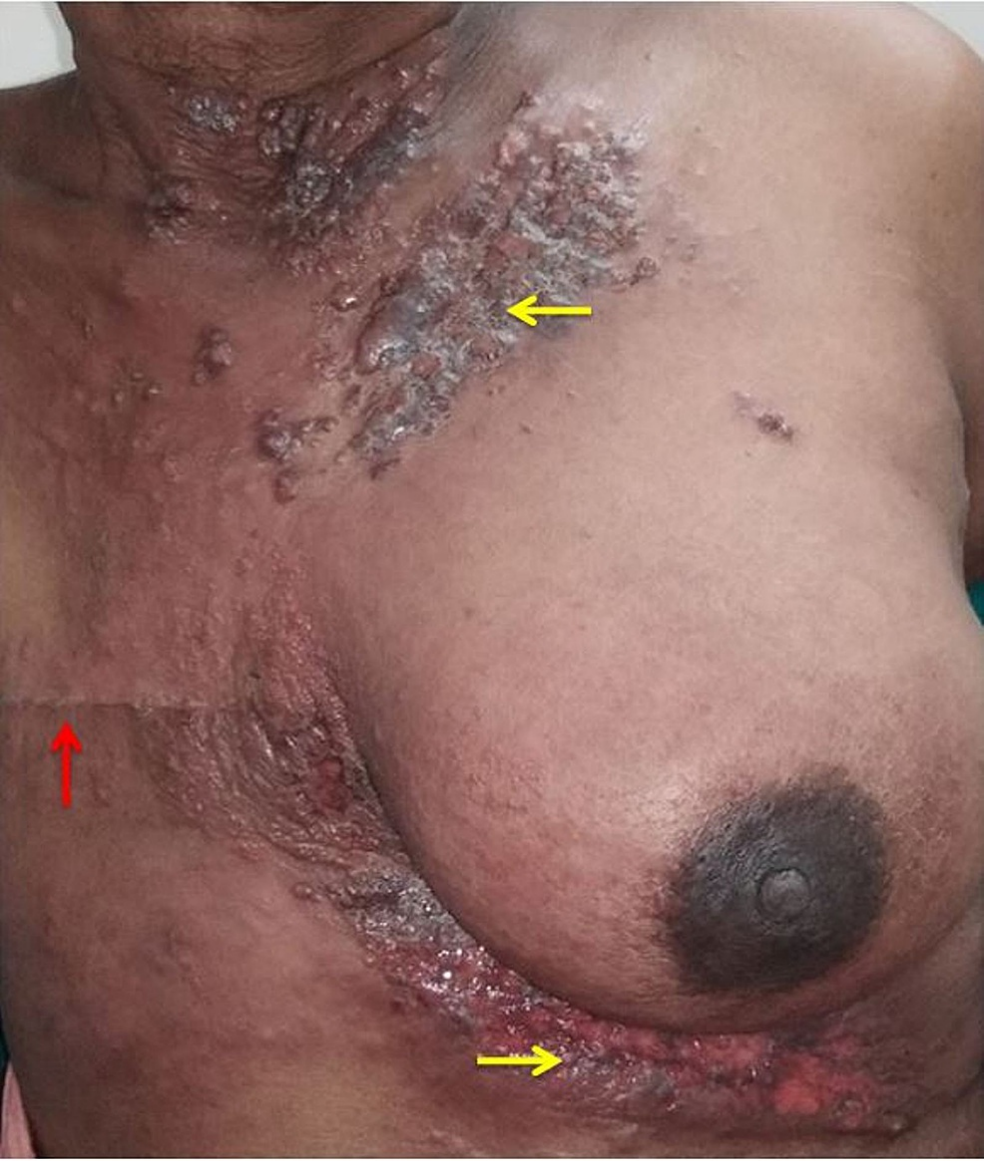
Erythematous plaques and nodules showing cutaneous recurrence on the contralateral side (yellow arrows) of the previous surgical site (red arrow).

A 52-year-old female presented to us with nodules over the chest wall. She had a history of carcinoma right breast diagnosed six months ago, for which she received four cycles of neoadjuvant chemotherapy and underwent right modified radical mastectomy in another hospital. The patient was advised to complete the remaining chemotherapy and radiotherapy postoperatively, but the patient did not follow up. After five months of surgery, she developed multiple papules and nodular lesions over the chest wall on the contralateral side without any itching, pain, or fever. On examination, the previous surgical scar was healthy. However, an erythematous nodular rash with induration was present over the left side of the neck and infraclavicular region and the left inframammary region (Figure 1).

**Figure 2:**
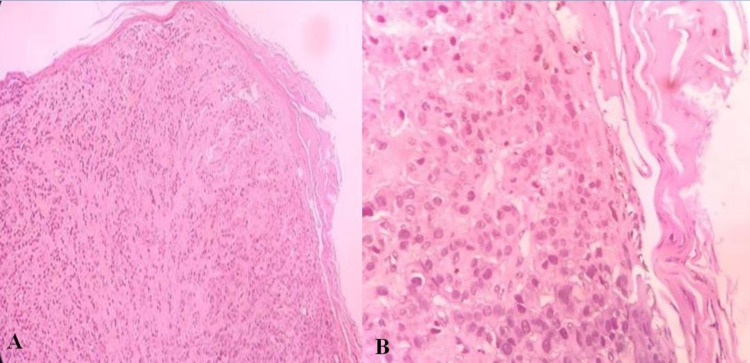
Histopathological image of the skin nodule showing epidermis and nests and sheets of tumor cells, trabecular arrangement in the dermis diagnostic of invasive ductal carcinoma; (A: 10x resolution and B: 40x resolution).

Viral etiology was ruled out. Skin biopsy was taken from the nodule and found to have nests and sheets of tumor cells, trabecular arrangement in the dermis, diagnostic of invasive ductal carcinoma (IDC) (Figure 2).

**Figure 3:**
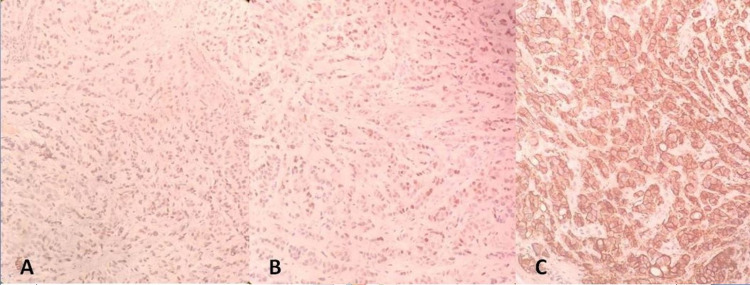
Immunohistochemistry image showing; A: Estrogen receptor (ER) positivity, B: Progesterone receptor (PR) positivity, and C: Human epidermal growth factor receptor 2 (HER2/neu) positive.

The tumor cells were estrogen receptor (ER), progesterone receptor (PR), and human epidermal growth factor receptor 2 (HER2/neu) positive on immunohistochemistry (Figure 3).

**Figure 4:**
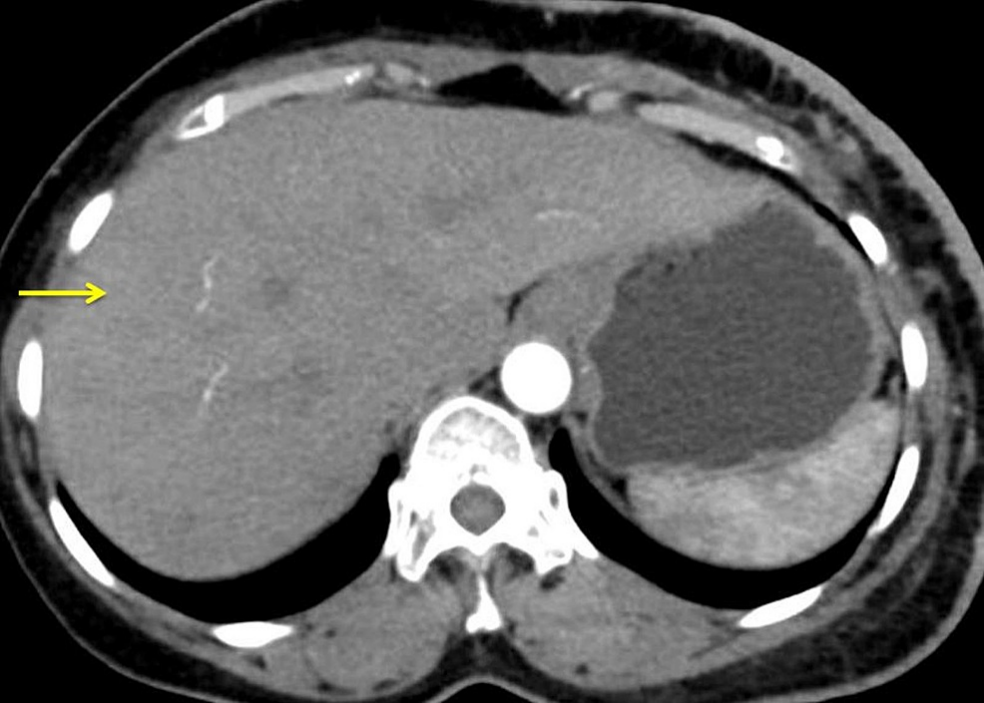
Computed tomography showing normal liver (arrow) without any evidence of distant metastasis.

A computed tomography (CT) scan was done, there was no evidence of distant metastasis (Figure 4).

The patient was discussed on the tumor board and was planned for palliative chemotherapy for a cutaneous recurrence of infiltrating ductal carcinoma of the breast. The patient was followed up for the next six months, and there was a decrease in the size of the cutaneous metastasis, and the patient was doing well.

## Discussion

Skin is not the first organ for metastasis from a visceral tumor. If the skin is involved, there is a very high chance that the tumor metastasis must have seeded in other vital organs such as the lungs, liver, and brain. The most common cancers that metastasize to the skin are melanoma, breast cancer, cancer of the nasal sinuses, laryngeal cancer, and oral cancer [1]. Carcinoma breast is the second most common malignancy in women. One of the common sites of metastasis from breast carcinoma is the skin [2]. Local isolated skin recurrence without concomitant metastatic disease after the mastectomy is usually rare. The most common sites involved are the chest wall, the abdomen, the back, the head and neck, and the upper extremities [3].

Early-onset tumors, incomplete tumor resection with positive margin status, presence of invasive occult focus, large multi quadrant tumors, comedo type, and high nuclear grade, and diffuse necrosis are reported as risk factors for local recurrence [4]. Skin involvement at locoregional recurrence increases the risk and incidence of distant metastases. Also, these patients have a high risk for distant metastases simultaneously or within two months of locoregional recurrence, compared with those patients without skin involvement at recurrence [5]. These local or regional cutaneous lesions occur due to direct or lymphatic spread from the primary tumor. Our patient had evidence of only cutaneous metastasis without evidence of any other metastasis.

The most common clinical presentation of cutaneous recurrence is either solitary or multiple erythematous infiltrating papules and nodules on the skin. This was the presentation in our patient as well. The rarer variants being targetoid lesions, carcinoma erysipeloides or inflammatory breast cancer, carcinoma en cuirasse, carcinoma telangiectatic, alopecia neoplastica, and zosteriform pattern [6]. The differential diagnoses of cutaneous lesions are varied. In a breast cancer patient, other possible aetiologies apart from cutaneous metastases include radiation dermatitis, other infectious processes such as erysipelas, herpes, cellulitis, or candidiasis [2].

The definitive diagnosis of cutaneous recurrence or metastasis secondary to breast carcinoma is a histopathological examination of a biopsy from the skin lesions. Histologically, they appear as atypical neoplastic cells arranged in small nests, islands, or cords in single-file within the collagen bundles of the dermis. The presence of invasion by malignant cells in the dermis similar to that of the primary tumor is the key to diagnosis. The immunohistochemical markers play a crucial role in prognosis and guiding the chemotherapy [3]. In our case, a biopsy was diagnostic of IDC.

Cutaneous metastases from breast cancer are incurable and are seen in advanced diseases. Therefore, treatment is primarily targeted towards local control with a multidisciplinary approach. While surgical resection and radiation therapy offer local control, antineoplastic therapy controls systemic disease [7]. However, in cases of diffuse skin involvement (as in our case), mainstream treatment would be chemotherapy, and surgery is the least resorted option. The prognosis in such cases depends on the tumor biology and tumor response to chemotherapy.

## Conclusions

Surgery alone does not offer a complete cure for breast cancer. A high index of clinical suspicion is required to diagnose cutaneous metastasis or recurrence in carcinoma breast. Such recurrences are usually associated with distant metastases and have an abysmal prognosis. Therefore, early detection and treatment should focus on treating these patients to improve survival rates with minimal morbidity.
